# Changes in Diet, Sleep, and Physical Activity Are Associated With Differences in Negative Mood During COVID-19 Lockdown

**DOI:** 10.3389/fpsyg.2020.588604

**Published:** 2020-09-02

**Authors:** Joanne Ingram, Greg Maciejewski, Christopher J. Hand

**Affiliations:** ^1^School of Education and Social Sciences, University of the West of Scotland, Paisley, United Kingdom; ^2^Department of Psychology, Glasgow Caledonian University, Glasgow, United Kingdom

**Keywords:** COVID-19, lockdown, diet, sleep, physical activity, alcohol, mood, mental health

## Abstract

The United Kingdom and Scottish governments instigated a societal lockdown in response to the COVID-19 pandemic. Subsequently, many experienced substantial lifestyle changes alongside the stresses of potentially catching the virus or experiencing bereavement. Stressful situations and poorer health behaviors (e.g., higher alcohol consumption, unhealthy diet, poorer sleep quality, physical inactivity) are frequently linked to poor mental health. Our objective was to examine changes in health behaviors and their relationship with negative mood during COVID-19 lockdown. We also considered associations between health behaviors and socio-demographic differences and COVID-19-induced changes. 399 participants completed a questionnaire asking about their personal situation and health behaviors during lockdown as well as a negative mood scale. The significance threshold for all analyses was α = 0.05. Poorer diet was linked to more-negative mood, and to changes to working status. Poorer sleep quality was linked with more-negative mood, and with ‘shielding’ from the virus. Being less physically active was related to more-negative mood and student status, whereas being more physically active was linked to having or suspecting COVID-19 infection within the household. Increased alcohol consumption was linked to living with children, but not to negative mood. Changes to diet, sleep quality, and physical activity related to differences in negative mood during COVID-19 lockdown. This study adds to reports on poor mental health during lockdown and identifies lifestyle restrictions and changes to health behaviors which may, to some extent, be responsible for higher negative mood. Our data suggests that it is advisable to maintain or improve health behaviors during pandemic-associated restrictions.

## Introduction

Lockdown conditions, arising from the developing COVID-19 situation, were introduced across the United Kingdom on 23rd March 2020. Residents of Scotland spent a total of 66 days under the most restrictive lockdown conditions, with these being eased to some extent on 28th May 2020^1^. The threat of contracting COVID-19 is an on-going stressor, though personal circumstances regarding work and home life may significant influence how individuals cope.

Restrictions and lifestyle changes during lockdown have been associated with poorer psychological wellbeing. Reduced social contact, feelings of isolation, and fear of contracting or spreading dangerous viruses have been historically linked to poor wellbeing (Bai et al., 2004; Hawryluck et al., 2004; Cacioppo and Hawkley, 2009; Chen and Feeley, 2014). Time spent in quarantine during previous disease outbreaks has been shown to have negative psychological effects (Brooks et al., 2020). Psychological symptoms (stress, anxiety, depression) have been shown to rise in tandem with time spent in lockdown (Ozamiz-Etxebarria et al., 2020). López-Bueno et al. (2020) proposed that poorer psychological wellbeing and mental health during COVID-19 lockdown is associated with health behaviors such as alcohol consumption, diet, sleep, and physical activity. We therefore examined the relationships between socio-demographic circumstances (e.g., living arrangements), COVID-induced changes in circumstances (e.g., changes to working arrangements, shielding), and changes in health behaviors. We also considered how changes to health behaviors related to negative mood, and we monitored changes in negative mood as lockdown restrictions eased.

Alcohol Change United Kingdom reported that individuals were drinking differently during the COVID-19 pandemic (Alcohol Change UK, 2020). In Scotland specifically, whilst 29% of drinkers surveyed reported that they had been drinking more-often since the beginning of lockdown, the same percentage reported they were drinking less-often (Alcohol Focus Scotland, 2020). Whilst there is evidence to suggest that alcohol-dependant individuals drink to alleviate negative emotional states (Koob, 2011), the relationship between emotional experience and alcohol in social drinkers is less-clearly defined (Sayette, 2017). Poor mental health has consistently been reported in abusive (Pottenger et al., 1978; Grant and Harford, 1995; Schuckit, 2006) and social drinkers (Birnbaum et al., 1983; Parker et al., 1987; Jones et al., 2007), and drinking alcohol has been associated with negative mood (Howland et al., 2010; Alford et al., 2020). In our study we focused specifically on *changes* to drinking behavior and examined links between these changes, socio-demographic and COVID-induced change circumstances, and negative mood.

Heightened life stress has been linked to unhealthy eating (Greeno and Wing, 1994; Ball and Lee, 2000), and stressed people are more likely to crave food high in energy, fats, and sugars (Steptoe et al., 1998; Wardle et al., 2000). Thus, it was expected that stressful changes and restrictions to daily life would lead to less-healthy eating habits. Research has already demonstrated poorer diet in children during COVID-19 lockdown (Pietrobelli et al., 2020), although other sources suggest that eating habits in adults have not changed or may even have improved for some (NL Times, 2020). Potential links between diets high in processed and sugary food and poor mental health have been reported (Jacka et al., 2010).

Stress has also been implicated in poor sleep quality and disrupted sleep (see Sanford et al., 2014 for a review). The COVID-19 pandemic has been linked to poor sleep quality in China (Xiao et al., 2020) and Italy (Casagrande et al., 2020), with over half of Italian respondents experiencing poor sleep quality. However, in Spain, the quality of respondents’ sleep seemed to improve as the lockdown progressed (López-Bueno et al., 2020). Critically, poor sleep quality has been linked to negative emotions and mood (see Baglioni et al., 2010 for a review).

Initial United Kingdom restrictions allowed leaving the house for exercise once per day, and facilities usually available for physical activities were closed. Lower rates of physical activity have been associated with feelings of social isolation (Robbins et al., 2018; Werneck et al., 2019), which may already be exacerbated by other restrictions of lockdown. It has been well-documented (see Landers and Arent, 2007 for a review) that physical activity is beneficial for mental health and wellbeing, and it would be assumed that any restrictions to physical activity would lead to poorer wellbeing. However, studies in Italy (Di Renzo et al., 2020) and Spain (López-Bueno et al., 2020) suggest that most individuals were increasing their physical activity levels during lockdown, which may be one of the ways of maintaining healthy behaviors and mitigating the negative impact of lockdown on mood and wellbeing.

Within the current study, we examined the associations between socio-demographic factors and COVID-induced changes, and health behaviors (changes in alcohol consumption, diet, sleep, and physical activity). We hypothesized that those who have experienced more restrictions or greater lifestyle changes would have increased incidence of change toward poor health behaviors (increased alcohol consumption, unhealthier diet, poorer sleep quality, and decreased physical activity). We considered how changes and differences in these behaviors related to mood. We predicted that those with changes toward poorer health behaviors would show higher negative mood. We also tracked changes in negative mood over three time points. At timepoint one, Scotland was under strict lockdown conditions where leaving the house was allowed for necessary work, to shop for essentials, and for unrestricted exercise. At timepoint two – two weeks later – restrictions had eased to allow meeting outside with one other household. At timepoint three – a further two weeks later – additional easing had taken place; meeting with two households was allowed outdoors and some people (e.g., those living alone) could form extended households. Restrictions to those in the shielding group (individuals who were completely isolating due to high risk) were also lifted around timepoint three. We hypothesized that this easing of lockdown restrictions would be associated with gradual improvements in mood.

## Materials and Methods

### Participants and Recruitment

Based on the work of Schönbrodt and Perugini (2018), anticipated small-to-medium effect sizes for *F*-tests, and an estimated Scottish adult population of approximately 4.3 million (National Records of Scotland, 2020), a target sample size between 250–430 was identified. 399 participants (56.4% female, 41.9% male, 1% non-binary, and 0.8% transgender) of various ages (range: 18–72 years, *M_*age*_* = 32.4, *SD_*age*_* = 11.4) completed the study. All were recruited through convenience sampling via Prolific Academic^2^; the sample consisted of the first 399 Prolific Academic users who identified as Scottish nationals/long-term residents (0.5–72 years; *M_*residence*_* = 29.0, *SD_*residence*_* = 12.2). 98.2% of the participants identified themselves as Caucasian, 1.5% as Asian, and 0.3% as Armenian. 315 of the 399 participants completed additional mood ratings in Week 3. 275 out of the 399 participants completed the ratings in Week 5.

Table 1 presents a summary of key demographics. Additionally, 27.3% and 8.5% of the sample had a child/children living in the same or a different household, respectively. 9.5% of the participants worked in the health/social care sector.

**TABLE 1:** Participant sample demographics.

**Sample**	***N***	***Mean Age***					
	**399**	**32.4 years (SD = 11.4)**					
**Gender-Sex**	*Female*	*Male*	*Non-Binary*	*Trans*			
	56.4%	41.9%	1%	0.8%			
**Location**	*Town*	*City*	*Suburbs*	*Village*	*Countryside*		
	32.3%	27.8%	21.1%	12.5%	6.0%		
**Sexuality**	*Heterosexual*	*Bisexual*	*Homosexual*	*Pansexual*	*Demisexual*	*Asexual*	*Queer*
	85.5%	7.5%	3.8%	0.8%	0.3%	0.3%	0.3%
**Relationship Status**	*Single*	*Married*	*Living with partner*	*In a relationship*	*Divorced*	*Separated*	
	29.3%	26.3%	23.3%	17.8%	1.8%	1.5%	
**Household**	*Living with partner only*	*Living with parents*	*Living with partner + children*	*Living alone*	*Other adult*	*Alone + child(ren)*	
	29.3%	26.8%	22.1%	12.0%	5.8%	4.0%	
**Student Status**	*Full-time*	*Part-time*	*Non-student*				
	21.8%	4.3%	73.9%				
**Employment**	*Working from home*	*Unemployed*	*Furloughed*	*Keyworker*	*Carer/Parent*	*Working Away*	
	35.3%	21.6%	19.0%	15.0%	5.0%	2.5%	
**Employment Change**	*Furloughed*	*Working from home*	*Unemployed*	*Keyworker*	*Carer/Parent*		
	46.9%	21.9%	17.2%	8.6%	2.3%		

#### Exposure to COVID-19

12.8% of participants suspected they had had COVID-19, 1% confirmed they had tested positive. 8.0% of participants reported they had lived with someone who suspected or had COVID-19 (half of whom suspected/had it themselves). 4.8% of participants had experienced bereavement due to COVID-19. 15.3% of participants reported that they were included in the shielding group, or a list of patients who were at high-risk of developing complications from COVID-19 due to pre-existing conditions (e.g., cancer, immunodeficiency).

### Materials and Procedure

The study received ethical approval from the School of Education and Social Sciences, University of the West of Scotland Ethics Committee, following World Medical Association (2013) and British Psychological Society (2014) protocols. Participants provided electronic informed consent prior to the study.

The study consisted of a questionnaire followed by a negative mood scale, both designed in Gorilla^3^. The first section of the questionnaire gathered the data presented in Table 1. The next section included yes-no questions on whether participants tested positive or suspected they had had COVID-19, lived with someone who tested positive or suspected they had had COVID-19, suffered bereavement as a result of COVID-19, and whether they were included in the shielding/vulnerable group. The third section included questions on how often participants would go out in public (and for what reasons), communicate with individuals from a different household (and how), and exercise in an average week during lockdown. We also included an 18-item multiple-choice checklist for participants to indicate how they spent time outside of working hours (e.g., exercise, reading books, gaming, online shopping), with an option to list additional activities.

#### Changes in Health Behaviors

In this section, participants rated changes to their alcohol consumption, diet, sleep quality, and physical activity during COVID-19 lockdown. A 5-point scale was used for all four (alcohol: 1 = “drinking a lot more”, 3 = “about the same”, 5 = “a lot less”; diet: 1 = “a lot more unhealthy”, 3 = “about the same”, 5 = “a lot more healthy”; sleep: 1 = “a lot worse”, 3 = “about the same”, 5 = “a lot better”; physical activity: 1 = “a lot less”, 3 = “about the same”, 5 = “a lot more”). The number and percentage of participants providing each rating can be seen in Table 2.

**TABLE 2:** Frequency and percentage of participants providing each health behavior rating.

**Alcohol**			**Diet**			**Sleep**			**Physical activity**		
**Change**	***N***	***%***	**Change**	***N***	***%***	**Change**	***N***	***%***	**Change**	***N***	***%***
A lot more	23	*5.8%*	A lot more unhealthy	51	*12.8%*	A lot worse	85	*21.3%*	A lot less	99	*24.8%*
A little more	118	*29.6%*	A little more unhealthy	112	*28.1%*	A little worse	124	*31.1%*	A little less	90	*22.6%*
Same	76	*19.0%*	Same	136	*34.1%*	Same	124	*31.1%*	Same	67	*16.8%*
A little less	41	*10.3%*	A little more healthy	75	*18.8%*	A little better	47	*11.8%*	A little more	94	*23.6%*
A lot less	60	*15.0%*	A lot more healthy	12	*6.3%*	A lot better	19	*4.8%*	A lot more	49	*12.3%*
Non-drinkers	81	*20.3%*	–	–	*–*	–	–	*–*	–	–	*–*

#### Negative Mood Score

We selected 10 negative items from Grove and Prapavessis’ (1992) abbreviated Profile of Mood State (POMS) scale, with two items taken from each of the five sub-dimensions of Confusion (*forgetful, unable to concentrate*), Tension (*anxious, uneasy*), Depression (*helpless, sad*), Fatigue (*exhausted, worn out*), and Anger (*angry, annoyed*). The abbreviated POMS scale has a mean subscale inter-correlation of 0.58 (0.53–0.67), mean subscale internal consistency (Cronbach’s *α*) of 0.80 (0.66–0.80), and clear validity (winner-loser differences *p* < 0.001). Total mood disturbance in the POMS is calculated by summing the scores on the negative subscales and subtracting the positive subscale; however, in the present study we used only the negative subscales. At each time point, participants in our study rated their mood on each of the 10 adjectives *at that point in time* using a 100-point slider scale, with higher scores indicating greater negative mood. For the current data set, a Cronbach’s *α* = 0.91 was observed for Negative Mood Score (NMS; *n*_*items*_ = 10), and the five subscales ranged from *α* = 0.69–0.89 (*M_α_* = 0.85).

### Data Analysis

Prior to inferential analysis, online survey responses were checked for completeness. Participants who identified as non-drinkers were excluded from analyses involving changes in alcohol consumption. A series of Pearson’s chi-square (χ^2^) analyses were conducted to explore the associations between socio-demographic factors and COVID-19 induced change on changes in health behaviors. A series of Spearman’s correlations were conducted to analyze relationships between changes in health behaviors – positive correlations represented ‘positive’ changes in the health behaviors (reduced alcohol consumption, healthier diet, better sleep, more physical activity). A series of univariate Analyses of Variance (ANOVAs) were conducted to explore the relationship between changes in health behaviors and isolation history on negative mood. All assumptions were met (e.g., expected frequencies, normality and equality of variance). Our significance threshold was α = 0.05, and corrections associated with follow-up comparisons are identified within the Results section.

## Results

### Socio-Demographic Circumstances, COVID-19 Induced Change, and Health Behaviors

Analyses are summarized in Table 3 below, and significant associations are explored in detailed below.

**TABLE 3:** Associations between socio-demographic circumstances, COVID-19 induced change, and health behaviors.

**Changes in**	**Alcohol Consumption**			**Diet**			**Sleep Quality**			**Physical Activity**		
	**χ^2^**	***df***	***p***	**χ^2^**	***df***	***p***	**χ^2^**	***df***	***p***	**χ^2^**	***df***	***p***
Child(ren) at home	**10.474**	**4**	**0.033**	5.913	4	0.206	2.894	4	0.576	3.916	4	0.418
Student status	13.279	8	0.103	12.480	8	0.131	10.987	8	0.202	**19.481**	**8**	**0.012**
Work status change	3.552	4	0.470	**9.768**	**4**	**0.045**	7.609	4	0.107	1.849	4	0.763
COVID-19 (Self)	0.981	4	0.913	1.166	4	0.884	1.697	4	0.791	0.926	4	0.921
COVID-19 (Household)	1.221	4	0.875	1.572	4	0.814	5.011	4	0.286	**12.055**	**4**	**0.017**
Shielding status	6.439	4	0.169	2.420	4	0.659	**13.200**	**4**	**0.010**	5.696	4	0.223
Vulnerability status	6.859	4	0.144	4.666	4	0.323	8.440	4	0.077	7.953	4	0.093
Self-isolation status	1.652	4	0.799	3.906	4	0.419	3.877	4	0.423	6.200	4	0.185

*Significant *p*-values (one-tailed) highlighted in bold.*

#### Alcohol

Analyses revealed that there was a significant association between whether or not the participants had children in their household during lockdown and their alcohol consumption [χ^2^(4) = 10.474, *p* = 0.033]. For those with children at home, there were more participants than expected drinking “a lot more” (10 vs. 5.8), and fewer than expected drinking “a lot less” (9 vs. 15.1); for child-less participants, there were fewer participants than expected drinking “a lot more” (12 vs. 16.2) and more participants than expected drinking “a little less” and “a lot less” (30 vs. 26.4 and 48 vs. 41.9, respectively).

#### Diet

Analyses revealed that there was a significant association between a change in work-status and a change in diet [χ^2^(4) = 9.768, *p* = 0.045]. For those whose work-status had changed, more participants than expected reported that their diet was “a little more unhealthy” (42 vs. 33.7), and fewer participants than expected maintained the same diet (28 vs. 40.9); for those participants reporting no change in work-status, fewer participants than expected reported that their diet was “a little more unhealthy” (61 vs. 69.3), and more participants than expected had maintained their pre-lockdown diet (97 vs. 84.1).

#### Sleep

Analyses revealed that there was a significant association between shielding status and lockdown changes in perceived sleep quality [χ^2^(4) = 13.200, *p* = 0.010]. With respect to shielding group participants, a greater number of participants than expected were sleeping “a lot worse” (22 vs. 12.4), and fewer shielding participants than expected maintained their pre-lockdown sleeping pattern (13 vs. 18.9); in contrast, fewer non-shielding participants than expected were sleeping “a lot worse” (54 vs. 63.6), and more non-shielding participants than expected had maintained their pre-lockdown sleeping pattern (103 vs. 97.1).

#### Physical Activity

Analyses revealed that there was a significant association between student-status and changes in activity levels [χ^2^(8) = 19.481, *p* = 0.012]. The pattern of observed vs. expected effects reveals that this is an association marked by general decline in activity. For full-time students, a greater number than expected reported being “a lot less” physically active (15 vs. 10.7), but fewer than expected reported being “a little less” active (17 vs. 21.6); for non-students, fewer participants than expected reported being “a lot less” physically active (29 vs. 32.5), but more than expected reported being “a little less” active than pre-lockdown (72 vs. 65.6).

Analyses also revealed an association between household COVID-19 infection status and activity level changes [χ^2^(4) = 12.055, *p* = 0.017]. For participants who had/suspected a case of COVID-19 infection within their households, more participants than expected reported being “a lot more” physically active than pre-lockdown (14 vs. 7.2), and less than expected reported being “a little more” active (3 vs. 6.3); for participants who had/suspected no cases in their household, more participants than expected had maintained their activity or were “a little more” active (58 vs. 54.3 and 77 vs. 73.7, respectively), but fewer than expected were “a lot more” active (77 vs. 83.8).

### Relationships Between Changes in Health Behaviors

Analyses revealed a moderate positive correlation between changes in alcohol consumption and diet (*r*_*S*_ = 0.299, *p* < 0.001), and a smaller positive relationship between alcohol consumption and physical activity level (*r*_*S*_ = 0.132, *p* = 0.006). There was no linear relationship between changes in alcohol consumption and changes in perceived sleep quality. There was a small positive correlation between changes to diet and perceived sleep quality (*r*_*S*_ = 0.187, *p* < 0.001), and a moderate positive correlation between changes in diet and changes in physical activity (*r*_*S*_ = 0.354, *p* < 0.001). Finally, there was a small positive correlation between changes in sleep quality and changes in physical activity (*r*_*S*_ = 0.191, *p* < 0.001).

### Health Behaviors and Negative Mood Score

**FIGURE 1:**
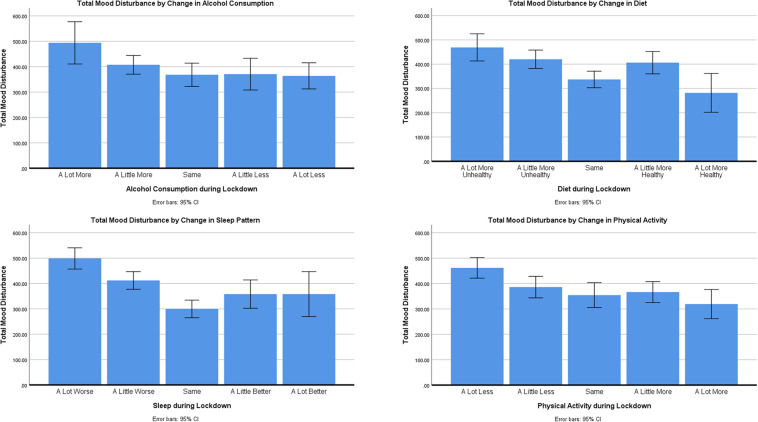
Mean negative mood score by health behavior.

ANOVAs across mood subscales are summarized in Supplementary Material 1 and Supplementary Table A1. Only total Negative Mood Score (NMS) will be discussed in detail. Post-hoc comparisons were conducted using Hochberg’s GT2 adjustment to account for differences in group sizes. Post-hoc comparisons are summarized in Supplementary Material 2 and Supplementary Table A2, and illustrated in Figure 1.

#### Alcohol

There was a no significant differences in NMS relating to changes in alcohol consumption [*F*(4,313) = 2.207, *p* = 0.068, *η*_*p*_^2^ = 0.027].

#### Diet

There were differences in NMS relating to changes in diet during lockdown [*F*(4,395) = 6.745, *p* < 0.001, *η*_*p*_^2^ = 0.064]. *Post-hoc* tests revealed that those with a much unhealthier diet during lockdown had greater NMS than those whose diets were the same (*p* = 0.001) or had improved a lot (*p* = 0.002). Those participants whose diet had only changed a little for the worse still reported greater NMS than those who had maintained the same diet (*p* = 0.014) and those who had a much-improved diet (*p* = 0.022) as before lockdown.

#### Sleep

There were clear differences In NMS relating to changes in perceived sleep quality during lockdown [*F*(4,395) = 13.831, *p* < 0.001, *η*_*p*_^2^ = 0.123]. *Post-hoc* tests revealed that those reporting that their sleep was much worse had greater NMS than all other groups (all *p’*s < 0.05), and those whose sleep was only a little worse during lockdown still had greater NMS than those whose sleep had been unaffected by lockdown (*p* < 0.001).

#### Physical Activity

There were difference in NMS relating to changes in physical activity during lockdown [*F*(4,395) = 5.321, *p* < 0.001, *η*_*p*_^2^ = 0.051]. *Post-hoc* tests revealed that those who were doing much less activity had greater NMS than those whose activity levels has stayed the same (*p* = 0.010) or improved a little (*p* = 0.013) or a lot (*p* = 0.001).

#### Isolation

There was a clear difference between whether or not participants had isolated or not during lockdown and NMS [*F*(1,397) = 8.818, *p* = 0.003, *η*_*p*_^2^ = 0.022]. Participants who had self-isolated reported higher NMS (*M*_*NMS*_ = 421.25, *SD*_*NMS*_ = 205.05) than those who had not isolated (*M*_*NMS*_ = 359.15, *SD*_*NMS*_ = 208.50).

We considered that there might have been an interaction between someone’s isolation status, health behaviors and NMS. We conducted a series of two-way between-subjects ANOVAs, and found no evidence for interactions between isolation status (yes, no) and changes in health behaviors on NMS (all *F*s < 1.763, all *p’*s > 0.136). These two-way ANOVAs returned consistent main effects of health behaviors and isolation group on NMS as the univariate ANOVAs summarized in Supplementary Material 1 and Supplementary Table A1.

### Changes in Mood Over Time

We examined whether the gradual easing of lockdown restrictions across the three time points (Weeks 1, 3, and 5) had any impact on participants’ mood states. Total NMS from 275 participants who completed all the time points were analyzed using an ANOVA with Time as a within-participants factor. The results showed that Time had a significant impact on NMS [*F*(2,548) = 31.99, *p* < 0.001, *η*_*p*_^2^ = 0.110]. We followed this up with pairwise comparisons (Bonferroni corrections) which revealed higher scores (i.e., more-negative mood) in Week 1 (*M* = 374.51, *SD* = 205.72) than Week 3 (*M* = 300.61, *SD* = 201.08; *p* < 0.001) but comparable scores in Weeks 3 and 5 (*M* = 306.29, *SD* = 215.96; *p* = 1). This suggests that participants’ mood states improved as a result of easing lockdown restrictions, albeit only in the early stages of the process.

## Discussion

This study has identified associations between socio-demographic factors, COVID induced change and health behaviors, and differences in negative mood dependent on changes to health behaviors. With the exception of alcohol and sleep, health behaviors were inter-related. Changes in diet, sleep, and physical activity had the clearest link to negative mood states.

Given previous links between mental health, mood, and alcohol (Birnbaum et al., 1983; Parker et al., 1987; Howland et al., 2010; Alford et al., 2020), it was predicted that those who were drinking more during lockdown would have higher negative mood scores. This prediction was not borne out in the analysis. Whilst changes in alcohol consumption were correlated with changes in other health behaviors (with the exception of sleep), it is not possible to associate better mood with a reduction in alcohol consumption during COVID-19 lockdown. There is some evidence to suggest that for social drinkers, under certain specific situations, alcohol can have a positive effect on emotion (Sayette, 2017), hence it is possible that under the short-term strict conditions alcohol consumption can improve the emotional state of some individuals. Overall, more participants in this study reported increases in drinking behavior in comparison to previously published data (Alcohol Focus Scotland, 2020).

Changes in work status were associated with changes in diet, those who had changed their work status due to the COVID-19 pandemic reported that their diet had been unhealthier. It was clear that those with an unhealthier diet had higher NMS. Poorer mental health has previously been linked to unhealthy diets (Sánchez-Villegas et al., 2009; Jacka et al., 2010; Parletta et al., 2017), and unhealthy diets have been linked to higher levels of life stress (Greeno and Wing, 1994; Ball and Lee, 2000). This suggests that COVID-19 related stress may have led to a change to less-healthy eating habits which could have led to development of more negative mood over lockdown. However, the current data set is limited as no conclusions can be drawn with respect to the causal relationship between negative mood and the health behaviors.

Shielding was the only COVID-19-related factor which was associated with changes in sleep quality. Those who were shielding during the COVID-19 lockdown were experiencing poorer sleep quality. As predicted, those who were sleeping more poorly during the lockdown were also found to have higher NMS. Links between stress and poor sleep (Sanford et al., 2014) are consistently reported, and NMS for those experiencing poorer sleep during this time would suggest complex interplay between stress, sleep disturbance, and mental health during COVID-19 lockdown.

Student status was associated with changes in physical activity, with those studying full-time seeing a greater reduction in their physical activity. Households where COVID-19 had been experienced or suspected were associated with a lot more physical activity, whilst households with no experience or suspicion of COVID-19 maintained physical activity or were a little more active. Those who were doing a lot less physical activity had significantly higher NMS than all other groups, with the exception of those doing a little less physical activity. It is clear that a reduction in the level of physical activity is associated with higher NMS. It is possible that this reduction is having an influence on participants’ mental health as being physically active has been shown to improve mood (Hartescu et al., 2015; Fritz and O’Connor, 2016), and is positively associated with mental wellbeing (Cerin et al., 2009). However, it is also possible that those experiencing high negative mood has reduced participants level of physical activity, which could support previously seen reduced levels of physical activity when feeling socially isolated (Robbins et al., 2018; Werneck et al., 2019). 35.9% of the participants reported having increased levels of physical activity during lockdown in comparison to 47.4% of the participants who had decreased their level of physical activity during lockdown. These results are in contrast to studies from Italy (Di Renzo et al., 2020) and Spain (López-Bueno et al., 2020) where participants generally reported an increase in activity during lockdown. This may reflect a trend for physical inactivity within Scotland (Murray, 2013). During and after lockdown periods, we recommend that physical activity – even within the home (da Cunha de Sá-Caputo et al., 2020; Mattioli et al., 2020) – and healthy diet should be promoted to combat sedentary behavior.

Lockdown conditions were associated with higher negative mood overall. This is compatible with previous research indicating that reduced, or the perception of reduced, social contact and health-based fears are related to poor wellbeing (Bai et al., 2004; Hawryluck et al., 2004; Cacioppo and Hawkley, 2009; Chen and Feeley, 2014) as well as previous research on psychological effects of quarantine or lockdown conditions (Brooks et al., 2020; Ozamiz-Etxebarria et al., 2020). Our results show that improvement in negative mood states were found quite quickly after the easing of lockdown conditions. Differences were found two weeks after lockdown conditions were relaxed. No further differences were found two weeks later, but this may reflect the relatively small differences in lockdown restrictions made during this time. Data on health behaviors was not collected at later timepoints for comparison.

Spending time in lockdown conditions has had a negative impact on mood, and this is in line with previously published work on the effects of COVID-19 lockdown on mental health (Ozamiz-Etxebarria et al., 2020). These results add to the growing body of literature on health and wellbeing during and after the COVID-19 pandemic and demonstrates that changes to health behaviors during this time may be to come extent responsible for poorer mood, anxiety and depression. However, the impact of these changes may be transient and persist primarily during the strictest lockdown conditions. The current study is somewhat limited in that potential co-variant health factors (i.e., pre-lockdown body-mass index, smoking / changes in smoking behavior) were not examined. Our sample – although large and representative – is somewhat limited in that only active internet users were recruited; COVID-19 lockdown was ongoing during recruitment; thus it was impossible to recruit outwith the online domain.

Future research should focus on establishing more specific details of the likely bidirectional causal relationship between poor mental health and changes in health behaviors during lockdown. Overall results suggest that those who had made small positive changes were demonstrating less negative mood. It is then suggested that were lockdown conditions to be reintroduced due to COVID-19 or another pandemic, wellbeing may be linked to making small improvements in diet, sleep and physical activity.

## Data Availability Statement

The raw data supporting the conclusions of this article will be made available by the authors, without undue reservation.

## Ethics Statement

The studies involving human participants were reviewed and approved by School of Education and Social Sciences, University of the West of Scotland Ethics Committee. The patients/participants provided their written informed consent to participate in this study.

## Author Contributions

JI performed initial research question, literature review, design, interpretation, and write-up. GM performed design, online survey/experiment building, data analysis, interpretation, and write-up. CH performed initial research question, design, data analysis, interpretation, write-up, and submission. All authors contributed to the article and approved the submitted version.

## Conflict of Interest

The authors declare that the research was conducted in the absence of any commercial or financial relationships that could be construed as a potential conflict of interest.

## Footnotes

1 With the exception of outdoor exercise which became unrestricted on 11th May 2020.

2 https://prolific.co

3 https://gorilla.sc/

## References

[B1] Alcohol Change UK (2020). *Drinking During Lockdown: Headline Finding.* Available online at: https://alcoholchange.org.uk/blog/2020/covid19-drinking-during-lockdown-headline-findings (accessed June 8, 2020).

[B2] Alcohol Focus Scotland (2020). *Scots Report Changing Drinking Patterns During Coronavirus Lockdown.* Available online at: https://www.alcohol-focus-scotland.org.uk/news/scots-report-changing-drinking-patterns-during-coronavirus-lockdown/ (accessed June 8, 2020).

[B3] Alford C. Martinkova Z. Tiplady B. Reece R. Verster J. C. (2020). The effects of alcohol hangover on mood performance assessed at home. *J. Clin. Med.* 9:1068. 10.3390/jcm9041068 32283738 PMC7231019

[B4] Baglioni C. Spiegelhelder K. Lombardo C. Riemann D. (2010). Sleep and emotions: a focus on insomnia. *Sleep Med. Rev.* 14 227–238. 10.1016/j.smrv.2009.10.007 20137989

[B5] Bai Y. Lin C.-C. Lin C.-Y. Chen J.-Y. Chue C.-M. Chou P. (2004). Survey of stress reactions among health care workers involved with the SARS outbreak. *Psychiatr. Serv.* 55 1055–1057. 10.1176/appi.ps.55.9.1055 15345768

[B6] Ball K. Lee C. (2000). Relationships between psychological stress, coping, and disordered eating: a review. *Psychol. Health* 14 1007–1035. 10.1080/08870440008407364 22175259

[B7] Birnbaum I. M. Taylor T. H. Parker E. S. (1983). Alcohol and sober mood state in female social drinkers. *Alcoholism* 7 362–368. 10.1111/j.1530-0277.1983.tb05483.x 6362459

[B8] British Psychological Society (2014). *Code of Human Research Ethics.* Leicester: BPS.

[B9] Brooks S. K. Webster R. K. Smith L. E. Woodland L. Wessely S. Greenberg N. (2020). The psychological impact of quarantine and how to reduce it: rapid review of the evidence. *Lancet* 395 912–920. 10.1016/S0140-6736(20)30460-8 32112714 PMC7158942

[B10] Cacioppo J. T. Hawkley L. C. (2009). Perceived social isolation and cognition. *Trends Cogn. Sci.* 13 447–454. 10.1016/j.tics.2009.06.005 19726219 PMC2752489

[B11] Casagrande M. Favieri F. Tambelli R. Forte G. (2020). The enemy who sealed the world: effects quarantine due to the COVID-19 on sleep quality, anxiety, and psychological distress in the Italian population. *Sleep Med.* [Preprint]. 10.1016/j.sleep.2020.05.011 32853913 PMC7215153

[B12] Cerin E. Leslie E. Sugiyama T. Owen N. (2009). Associations of multiple physical activity domains with mental well-being. *Ment. Health Phys. Act.* 2 55–64. 10.1016/j.mhpa.2009.09.004

[B13] Chen Y. Feeley T. H. (2014). Social support, social strain, loneliness, and well-being among older adults: an analysis of the health and retirement study. *J. Soc. Pers. Relat.* 31 141–161. 10.1177/0265407513488728

[B14] da Cunha de Sá-Caputo D. Taiar R. Seixas A. Sanudo B. Sonza A. Bernardo-Filho M. A. (2020). Proposal of physical performance tests adapted as home workout options during the COVID-19 Pandemic. *Appl. Sci.* 10:4755 10.3390/app10144755

[B15] Di Renzo L. Gualtieri P. Pivari F. Soldati L. Attinà A. Cinelli G. (2020). Eating habits and lifestyle changes during COVID-19 lockdown: an Italian Survey. *J. Transl. Med.* 18:229. 10.1186/s12967-020-02399-5 32513197 PMC7278251

[B16] Fritz K. M. O’Connor P. J. (2016). Acute exercise improves mood and motivation in young men with ADHD symptoms. *Med. Sci. Sports Exerc.* 48 1153–1160. 10.1249/mss.0000000000000864 26741120

[B17] Grant B. F. Harford T. C. (1995). Comorbidity between DSM-IV alcohol use disorders and major depression: results of a national survey. *Drug Alcohol Depend.* 39 197–206. 10.1016/0376-8716(95)01160-4 8556968

[B18] Greeno C. Wing R. (1994). Stress-induced eating. *Psychol. Bull.* 115 444–464. 10.1037/0033-2909.115.3.444 8016287

[B19] Hartescu I. Morgan K. Stevinson C. D. (2015). Increased physical activity improves sleep and mood outcomes in inactive people with insomnia: a randomized controlled trial. *J. Sleep Res.* 24 526–534. 10.1111/jsr.12297 25903450

[B20] Hawryluck L. Gold W. L. Robinson S. Pogorski S. Galea S. Styra R. (2004). SARA control and psychological effects of quarantine, Toronto, Canada. *Emerg. Infect. Dis.* 10 1206–1212. 10.3201/eid1007.030703 15324539 PMC3323345

[B21] Howland J. Rohsenow D. J. Greece J. A. Littlefield C. A. Almeida A. Heeren T. (2010). The effects of binge drinking on college students’ next-day academic test-taking performance and mood state. *Addiction* 105 655–665. 10.1111/j.1360-0443.2009.02880.x 20403018 PMC2859622

[B22] Jacka F. N. Pasco J. A. Mykletun A. Williams L. J. Hodge A. M. O’Reilly S. L. (2010). Association of Western and traditional diets with depression and anxiety in women. *Am. J. Psychiatr.* 167 305–311. 10.1176/appi.ajp.2009.09060881 20048020

[B23] Jones F. O’Connor D. B. Conner M. McMillan B. Ferguson E. (2007). Impact of daily mood, work hours, and iso-strain variables on self-reported health behaviors. *J. Appl. Psychol.* 92 1731–1740. 10.1037/0021-9010.92.6.1731 18020809

[B24] Koob G. F. (2011). “Theoretical frameworks and mechanistic aspects of alcohol addiction: alcohol addiction as a reward deficit disorder,” in *Behavioral Neurobiology of Alcohol Addiction Current Topics*, Vol. 13 eds Sommer W. Spanagel R. (Berlin: Springer), 3–30. 10.1007/978-3-642-28720-6_129 PMC3448980 21744309

[B25] Landers D. M. Arent S. M. (2007). “Physical activity and mental health,” in *Handbook of Sport Psychology*, eds Tenenbaum G. Eklund R. C. (New York, NY: John Wiley and Sons Inc), 469–491.

[B26] López-Bueno R. Calatayud J. Casaña J. Casajús J. A. Smith L. Tully M. A. (2020). COVID-19 Confinement and health risk behaviors in Spain. *Front. Psychol.* 11:1426. 10.3389/fpsyg.2020.01426 32581985 PMC7287152

[B27] Mattioli A. V. Ballerini Puviani M. Nasi M. (2020). COVID-19 pandemic: the effects of quarantine on cardiovascular risk. *Eur. J. Clin. Nutr.* 74 852–855. 10.1038/s41430-020-0646-z 32371988 PMC7199203

[B28] Murray A. (2013). Physical inactivity – Getting Scotland on the move. *Br. J. Sport. Med.* 47 191–192. 10.1136/bjsports-2012-091122 22554847

[B29] National Records of Scotland (2020). *Mid-year Population Estimates Scotland, Mid-2019.* Available online at: https://www.nrscotland.gov.uk/files//statistics/population-estimates/mid-19/mid-year-pop-est-19-report.pdf (accessed August 8, 2020).

[B30] Ozamiz-Etxebarria N. Mondragon N. I. Santamaria M. D. Gorrotxategi M. P. (2020). Psychological symptoms during the two stages of lockdown in response to the COVID-19 outbreak: an investigation in a sample of citizens in Northern Spain. *Front. Psychol.* 11:1491. 10.3389/fpsyg.2020.01491 32625157 PMC7314923

[B31] Parker D. A. Parker E. S. Harford T. C. Farmer G. C. (1987). Alcohol use and depression symptoms among employed men and women. *Am. J. Public Health* 77 704–717. 10.2105/AJPH.77.6.704 3578617 PMC1647067

[B32] Parletta N. Zarnowiecki D. Cho J. Wilson A. Bogomolova S. Villani A. (2017). A Mediterranean-style dietary intervention supplemented with fish oil improves diet quality and mental health in people with depression: a randomized controlled trial (HELFIMED). *Nutr. Neurosci.* 22 1–14. 10.1080/1028415X.2017.1411320 29215971

[B33] Pietrobelli A. Pecoraro L. Ferruzzi A. Heo M. Faith M. Zoller T. (2020). Effects of COVID-19 lockdown on lifestyle behaviours in children with obesity living in Verona, Itlay: a longitudinal study. *Obesity* 10.1002/oby.22861 [Epub ahead of print]. 32352652 PMC7267384

[B34] Pottenger M. McKernon J. Patrie L. E. Weissman M. M. Ruben H. L. Newberry P. (1978). The frequency and persistence of depressive symptoms in the alcohol abuser. *J. Nervous Ment. Dis.* 166 562–570. 10.1097/00005053-197808000-00003 681916

[B35] Robbins L. M. Hill K. D. Finch C. F. Clemson L. Haines T. (2018). The association between physical activity and social isolation in community-dwelling older adults. *Aging Ment. Health* 22 175–182. 10.1080/13607863.2016.1242116 27736192

[B36] Sánchez-Villegas A. Delgado-Rodrígues M. Alonso A. Schlatter J. Lahortiga F. Majem L. S. (2009). Association of the mediterranean dietary pattern with incidence of depression: the Seguimiento Universidad de Navarra/University of Navarra follow-up (SUN) cohort. *Arch. Gen. Psychiatry* 66 1090–1098. 10.1001/archgenpsychiatry.2009.129 19805699

[B37] Sanford L. D. Suchecki D. Meerlo P. (2014). “Stress, arousal and sleep,” in *Sleep, Neuronal Plasticity and Brain Function Current Topics in Behavioral Neurosciences*, Vol. 25 eds Meerlo P. Benca R. Abel T. (Berlin: Springer), 379–410. 10.1007/7854_2014_314 24852799

[B38] Sayette M. A. (2017). The effects of alcohol on emotion in social drinkers. *Behav. Res. Ther.* 88 76–89. 10.1016/j.brat.2016.06.005 28110679 PMC5724975

[B39] Schönbrodt F. D. Perugini M. (2018). At what sample size do correlations stabilize? *J. Res. Personal.* 47 609–612. 10.1016/j.jrp.2013.05.009

[B40] Schuckit M. A. (2006). Comorbidity between substance use disorders and psychiatric conditions. *Addiction* 101 (Suppl. 1), 76–88. 10.1111/j.1360-0443.2006.01592.x 16930163

[B41] Steptoe A. Lipsey Z. Wardle J. (1998). Stress, hassles, and variations in alcohol consumption, food choice, and physical exercise: a diary study. *Br. J. Health Psychol.* 3 51–63. 10.1111/j.2044-8287.1998.tb00555.x

[B42] NL Times (2020). *Most Dutch Didn’t Change Their Eating Habits in Lockdown.* Available online at: https://nltimes.nl/2020/05/07/dutch-didnt-change-eating-habits-lockdown (accessed June 9, 2020).

[B43] Wardle J. Steptoe A. Oliver G. Lipsey Z. (2000). Stress, dietary restraint, and food intake. *J. Psychosom. Res.* 48 195–202. 10.1016/S0022-3999(00)00076-3 10719137

[B44] Werneck A. O. Collings P. J. Barboza L. L. Stubbs B. Silva D. R. (2019). Associations of sedentary behaviors and physical activity with social isolation in 100,839 school students: the Brazilian Scholar Health survey. *Gen. Hosp. Psychiatry* 59 7–13. 10.1016/j.genhosppsych.2019.04.010 31054464

[B45] World Medical Association (2013). Declaration of Helsinki: ethical principles for medical research involving human subjects. *JAMA* 310 2191–2194. 10.1001/jama.2013.281053 24141714

[B46] Xiao H. Zhang Y. Kong D. Li S. Yang N. (2020). The effects of social support on sleep quality of medical staff treating patients with coronavirus disease 2019 (COVID-19) in January and February 2020 in China. *Med. Sci. Monit.* 26 e923549-1–e923549-8. 10.12659/MSM.923549 32132521 PMC7075079

