# Corrigendum: Changes in Diet, Sleep, and Physical Activity Are Associated With Differences in Negative Mood During COVID-19 Lockdown

**DOI:** 10.3389/fpsyg.2020.605118

**Published:** 2020-10-21

**Authors:** Joanne Ingram, Greg Maciejewski, Christopher J. Hand

**Affiliations:** ^1^School of Education and Social Sciences, University of the West of Scotland, Paisley, United Kingdom; ^2^Department of Psychology, Glasgow Caledonian University, Glasgow, United Kingdom

**Keywords:** COVID-19, lockdown, diet, sleep, physical activity, alcohol, mood, mental health

In the original article, there was an error. An inaccurate statement was made in the Funding statement.

A correction has been made to Funding. The corrected statement reads as follows:

Funding was awarded as part of a portfolio submission by the University of the West of Scotland to the Chief Scientist Office, part of the Scottish Government Health and Social Care Directorate.

The authors apologize for this error and state that this does not change the scientific conclusions of the article in any way. The original article has been updated.

