# Supplementary material for: Changes in Diet, Sleep, and Physical Activity Are Associated With Differences in Negative Mood During COVID-19 Lockdown
Source: Front Psychol. 2020 Sep 2;11:588604. doi: 10.3389/fpsyg.2020.588604 (PMC7492645; doi:10.3389/fpsyg.2020.588604)
Supplement: Supplementary file 1 [file Table_1.docx]

**Supplementary Material 1.** Table A1. Summary of ANOVAs across Health Behaviours, Self-Isolation status, and Mood Measures

|  |  | **Alcohol** | | | **Diet** | | | **Sleep** | | | | **Physical Activity** | | | | **Isolation** | | |
| --- | --- | --- | --- | --- | --- | --- | --- | --- | --- | --- | --- | --- | --- | --- | --- | --- | --- | --- |
|  | **Measure** | *F* | *p* | *η_p_^2^* | *F* | *p* | *η_p_^2^* | *F* | *p* | *η_p_^2^* | *F* | | *p* | *η_p_^2^* | *F* | | *p* | *η_p_^2^* |
| Total | Negative Mood Score | 2.280 | .061 |  | 6.745 | **<.001** | .064 | 13.831 | **<.001** | .123 | 5.321 | | **<.001** | .051 | 8.818 | | .**003** | .022 |
| Subscale | Confusion | 2.207 | .068 |  | 3.931 | **.004** | .038 | 7.188 | **<.001** | .068 | 5.323 | | **<.001** | .051 | 6.837 | | .099 |  |
| Subscale | Tension | 2.063 | .086 |  | 3.770 | **.005** | .037 | 8.525 | **<.001** | .079 | 2.453 | | **.045** | .024 | 3.441 | | .064 |  |
| Subscale | Depression | 2.022 | .091 |  | 5.206 | **<.001** | .050 | 9.013 | **<.001** | .084 | 5.725 | | **<.001** | .055 | 5.023 | | .**026** | .012 |
| Subscale | Fatigue | 2.135 | .076 |  | 7.577 | **<.001** | .071 | 16.911 | **<.001** | .146 | 6.145 | | **<.001** | .059 | 6.262 | | .**013** | .016 |
| Subscale | Anger | 1.114 | .350 |  | 2.394 | **.050** | .24 | 5.740 | **<.001** | .055 | <1 | |  |  | 7.599 | | .**006** | .019 |

Note. Effect sizes (*η*_p_^2^) are only provided when *p*≤.05. Degrees of freedom: Alcohol (4,313), Diet/Sleep/Physical Activity (4,395), Isolation (1,397).
