# Supplementary material for: Changes in Diet, Sleep, and Physical Activity Are Associated With Differences in Negative Mood During COVID-19 Lockdown
Source: Front Psychol. 2020 Sep 2;11:588604. doi: 10.3389/fpsyg.2020.588604 (PMC7492645; doi:10.3389/fpsyg.2020.588604)
Supplement: Supplementary file 2 [file Table_2.docx]

**Supplementary Material 2.** Table A2. Post hoc comparisons of Health Behaviour changes and total Negative Mood Score (NMS)

| **Alcohol** | | Diff | *p* | **Diet** | | Diff | *P* | **Sleep** | | Diff | *p* | **Physical Activity** | | Diff | *p* |
| --- | --- | --- | --- | --- | --- | --- | --- | --- | --- | --- | --- | --- | --- | --- | --- |
| ++ More | + More | 26 | .141 | ++ Unhealthy | + Unhealthy | 49 | .813 | ++ Worse | + Worse | 87 | .019 | ++ Less | + Less | 76 | .109 |
|  | = Same | 29 | .103 |  | = Same | **132** | **.001** |  | **= Same** | **199** | **<.001** |  | **= Same** | **107** | **.010** |
|  | + Less | 32 | .095 |  | + Healthy | 63 | .604 |  | **+ Better** | **141** | **.001** |  | **+ More** | **95** | **.013** |
|  | ++ Less | 32 | .056 |  | ++ Healthy | **187** | **.002** |  | **++ Better** | **141** | **.050** |  | **++ More** | **143** | **.001** |
| + More | = Same | 3 | >.999 | + Unhealthy | = Same | **83** | **.014** | + Worse | **= Same** | **112** | **<.001** | + Less | = Same | 32 | .983 |
|  | + Less | 6 | .999 |  | + Healthy | 14 | >.999 |  | + Better | 54 | .677 |  | + More | 20 | .999 |
|  | ++ Less | 6 | .996 |  | ++ Healthy | **138** | **.022** |  | ++ Better | 54 | .954 |  | ++ More | 67 | .492 |
| = Same | + Less | 3 | >.999 | = Same | + Healthy | 70 | .167 | = Same | + Better | 59 | .569 | = Same | + More | 12 | >.999 |
|  | ++ Less | 3 | >.999 |  | ++ Healthy | 55 | .906 |  | ++ Better | 58 | .924 |  | ++ More | 35 | .988 |
| + Less | ++ Less | 0 | >.999 | + Healthy | ++ Healthy | 124 | .080 | + Better | ++ Better | 0 | >.999 | + More | ++ More | 47 | .876 |

Note. Diff = mean difference, rounded to whole integers. Significant p-values highlighted in **bold**.
